# Communicating Intent of Automated Vehicles to Pedestrians

**DOI:** 10.3389/fpsyg.2018.01336

**Published:** 2018-08-07

**Authors:** Azra Habibovic, Victor Malmsten Lundgren, Jonas Andersson, Maria Klingegård, Tobias Lagström, Anna Sirkka, Johan Fagerlönn, Claes Edgren, Rikard Fredriksson, Stas Krupenia, Dennis Saluäär, Pontus Larsson

**Affiliations:** ^1^RISE Research Institutes of Sweden, Gothenburg, Sweden; ^2^Volvo Cars Group, Gothenburg, Sweden; ^3^Autoliv AB, Vårgårda, Sweden; ^4^Scania AB, Södertälje, Sweden; ^5^Volvo Group AB, Gothenburg, Sweden

**Keywords:** automated vehicle, pedestrian, interaction, external interface, intent, communication, negotiation

## Abstract

While traffic signals, signs, and road markings provide explicit guidelines for those operating in and around the roadways, some decisions, such as determinations of “who will go first,” are made by implicit negotiations between road users. In such situations, pedestrians are today often dependent on cues in drivers’ behavior such as eye contact, postures, and gestures. With the introduction of more automated functions and the transfer of control from the driver to the vehicle, pedestrians cannot rely on such non-verbal cues anymore. To study how the interaction between pedestrians and automated vehicles (AVs) might look like in the future, and how this might be affected if AVs were to communicate their intent to pedestrians, we designed an external vehicle interface called automated vehicle interaction principle (AVIP) that communicates vehicles’ mode and intent to pedestrians. The interaction was explored in two experiments using a Wizard of Oz approach to simulate automated driving. The first experiment was carried out at a zebra crossing and involved nine pedestrians. While it focused mainly on assessing the usability of the interface, it also revealed initial indications related to pedestrians’ emotions and perceived safety when encountering an AV with/without the interface. The second experiment was carried out in a parking lot and involved 24 pedestrians, which enabled a more detailed assessment of pedestrians’ perceived safety when encountering an AV, both with and without the interface. For comparison purposes, these pedestrians also encountered a conventional vehicle. After a short training course, the interface was deemed easy for the pedestrians to interpret. The pedestrians stated that they felt significantly less safe when they encountered the AV without the interface, compared to the conventional vehicle and the AV with the interface. This suggests that the interface could contribute to a positive experience and improved perceived safety in pedestrian encounters with AVs – something that might be important for general acceptance of AVs. As such, this topic should be further investigated in future studies involving a larger sample and more dynamic conditions.

## Introduction

The latest technological advancements in the road vehicle domain suggest that vehicles having advanced robotic characteristics will become an integral part of our future transportation system (Anderson et al., 2014). More specifically, advanced driver support systems such as adaptive cruise control (ACC), automatic emergency braking (AEB), and pedestrian safety are already on the market, while automated driving systems that assume either partial or full authority from vehicle drivers are under development. Such vehicles are expected to bring many benefits to society, including improved safety, reduced congestion, lower levels of emissions, higher productivity, and greater mobility. However, to facilitate these benefits and ensure large-scale introduction, there are some challenges that must be overcome.

One of the challenges is how automated vehicles (AVs) should interact with other road users (agents) in their vicinity, thereby contributing to a safe traffic system and gaining public acceptance. In the future, AVs will be expected to coexist with conventional vehicles as well as with vulnerable road users such as pedestrians and cyclists. Consequently, some of the current interactions between road users may be impaired, while some others may be improved. These interactions are currently largely unexplored as the focus in the research community is primarily on tackling challenges associated with the human–machine interaction (HMI) inside an AV, such as the driver’s ability to seize control from the vehicle.

Indeed, our earlier study on interactions between pedestrians and AVs (Malmsten Lundgren et al., 2017) shows that communication needs may change when highly and fully AVs are introduced in the traffic system. More specifically, it shows that pedestrians may gain from knowing the mode and intent of AVs. That is in line with other studies from human–robotic interaction suggesting that mutual understanding of each other’s intent is crucial for safe and pleasant interactions (Klein et al., 2004). A way to facilitate mutual understanding is therefore to clearly communicate the AV’s own intent to the surrounding agents.

In this study, we further investigate the communication needs of pedestrians in interactions with AVs by exploring a technical means for communication. The automated vehicle interaction principle (AVIP) is an external vehicle interface that communicates the mode and intent of AVs to pedestrians (and other road users), and thereby functions as a replacement for the current driver–pedestrian interaction. Rather than being a concept to inspire future vehicle design, the AVIP interface is used as a research tool to study the effect of intent communication. In particular, the AVIP is used to investigate the effect of intent communication on pedestrians’ perceived safety. The study is based on two experiments where a Wizard of Oz (WOZ) approach was used to simulate automated driving (Molin, 2014). The first experiment was carried out at a zebra crossing. While it focused on assessing the usability of the interface, it revealed also initial information on pedestrians’ emotions and perceived safety when encountering an AV both with and without the interface in a situation where vehicles are legally required to yield to pedestrians. In addition, it informed the design of the second experiment that was carried out at a parking lot where the yielding rules are less clear and more negotiable. The second experiment focused on the assessment of pedestrians’ perceived safety when encountering an AV with/without the interface. For comparison purposes, these pedestrians encountered a conventional vehicle as well.

The rest of this paper describes the background of the study, the AVIP interface, and the evaluation methodology. Finally, the major results from the evaluations are presented and discussed.

## Background

### Interactions Between Conventional Vehicles and Pedestrians

**FIGURE 1:**
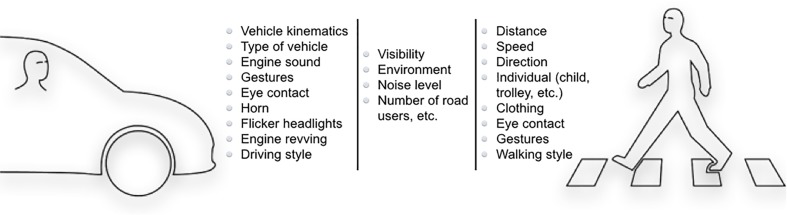
There are several cues that are important in the current vehicle–pedestrian interaction.

To achieve a safe and pleasant interaction, road users involved need to have a similar interpretation of the situation. If this is not the case, and road users differ in their interpretation or awareness of the situation, breakdowns in the interaction and conflicts are likely to occur (Endsley, 1995). Indeed, misinterpretation is one of the most common causation factors in pedestrian incidents and accidents (Habibovic and Davidsson, 2012). How pedestrians and vehicles interact is, however, still not fully understood. It is known that these interactions are complex and often affected by various factors (**Figure 1**) such as: vehicle speed and time to collision (Várhelyi, 1998; Sun et al., 2015; Schneemann and Gohl, 2016), traffic density and size of the gap between the vehicles (Wang et al., 2010), road features such as geometry and signs (Knoblauch et al., 1996), weather and light conditions (Sun et al., 2015), crossing speed (Knoblauch et al., 1996), presence and behavior of other road users (Rosenbloom, 2009; Zhou et al., 2009), demographics of drivers and pedestrians (Oxley et al., 2005; Lobjois and Cavallo, 2007; Tom and Granié, 2011), as well as their experiences, knowledge, motivations, and cognitive state (Mwakalonge et al., 2015). In addition, expectations and feelings of safety or insecurity affect the way the interactions develop (Zhou et al., 2009; Malmsten Lundgren et al., 2017; Sucha et al., 2017).

In some traffic situations, especially at low speeds and when there are ambiguities and negotiation is needed, road users also use non-verbal communication to clarify their intentions. Sucha et al. (2017) found that pedestrians’ decisions to cross and feeling of safety are affected by various signals given by the driver such as eye contact, waving a hand, posture, and flashing lights. They noticed that 84% of pedestrians sought eye contact with drivers. Schmidt and Färber (2009) found that pedestrians who want to cross the street look at the approaching driver to get “acknowledgment” from the driver, i.e., if the driver returns the eye contact, pedestrians assume that they have been seen and that they have achieved mutual understanding. The importance of visual search is also evident from the study by Luoma and Peltola (2013) demonstrating that 75% of pedestrians walked facing toward rather than with traffic. This same behavior correlated with lower fatality risk in historic data. Similar conclusions were drawn by Rasouli et al. (2017) where it was shown that the most prominent signal to transmit pedestrians’ crossing intention is looking (90%), or glancing (10%), toward the oncoming traffic. In their study, Schneemann and Gohl (2016) found that when pedestrians interact with vehicles, they tend to rely on eye contact with the driver in low speeds, while in faster speed zones, they base their decisions more on the behavior of the vehicle.

Studies conducted on the effects of non-verbal signals that pedestrians use to communicate with drivers provide some further explanation of the nature of the interaction between these road users. Guéguen et al. (2015) found that pedestrian eye contact is one of the factors having a strong influence on driver behavior. Without eye contact, about 55% of the drivers did not stop for the pedestrian, while about 68% of the drivers stopped when the pedestrian was seeking eye contact. A positive effect of pedestrians’ eye contact and/or other gestures (e.g., hand waving, leg movements, and smile) is also demonstrated in terms of increased time to collision and decreased severe braking by drivers (Ren et al., 2016), and increased drivers’ yielding behavior (Nasar, 2003; Crowley-Koch et al., 2011; Zhuang and Wu, 2014; Guéguen et al., 2016). Schmidt and Färber (2009) showed that participants were unable to correctly evaluate pedestrians’ crossing intentions based only on their trajectories, suggesting that parameters of body language are valuable cues.

This review demonstrates that non-verbal mutual communication between pedestrians and drivers is an important, yet relatively understudied, aspect of safe interactions in the current traffic system. The non-verbal communication signals a road user’s intent, telling or confirming to other road users in the vicinity what is about to happen next. As such, non-verbal communication between drivers and pedestrians is often of a proactive nature. Its importance is especially manifested in low speeds where negotiations are crucial, which is also the reason that our experiments capture two of such situations (a zebra crossing and a parking lot).

### Interactions Between Automated Vehicles and Pedestrians

Automated vehicles with high levels of automation (see SAE International, 2016) able to operate in urban and suburban environments are generally not available on the market yet. Consequently, interactions between them and pedestrians are largely unexplored, but a limited number of examples exist. A recent study by Rothenbucher et al. (2016), where 67 interactions between a (seemingly) fully AV and pedestrians were investigated, showed that pedestrians generally adhered to existing interaction patterns with vehicles unless the vehicle was behaving recklessly (e.g., decelerating late). Our own study, on the other hand, suggests that the introduction of AVs in the urban context may lead to a notable change in how pedestrians experience AVs compared to conventional vehicles (Habibovic et al., 2016; Malmsten Lundgren et al., 2017). The pedestrian participants rated eye contact with the driver as promoting calm interaction, while apparent driver distraction (e.g., talking on the phone and reading newspaper) led to pedestrian stress and ratings indicating an unpleasant interaction. Also, the pedestrians stated that they would look for confirmation from the “driver,” and that future AVs should clearly inform pedestrians about their mode and intent. They implied that knowing the mode of the vehicle would allow them early to distinguish what type of vehicle they are encountering, and enable them to align their expectations. Similarly, knowing the intentions of the vehicle would eliminate possible ambiguities due to the lack of communication with the “driver.” This is in line with the conclusions presented by Merat et al. (2016) and Böckle et al. (2017) where interactions between fully automated shuttles and pedestrians have been investigated in real-world traffic and using virtual reality, respectively.

Based on these somewhat contradictory findings, it might be possible that interactions between pedestrians and AVs are ambiguous, due to the lack of non-verbal communication that pedestrians are currently used to having with drivers of conventional vehicles. The changes in pedestrians’ experiences were typically linked to the changing role of the driver, who becomes a more passive participant in the interaction. With the transfer of control from the driver to the vehicle, pedestrians will not be able to rely on cues in driver behavior anymore (Stanciu et al., 2017). This could, in turn, lead to misinterpretation of an AV’s intent and increase the risk of unpleasant encounters.

### The Value of Showing Intentions

Given that AVs will be involved in social interactions with pedestrians at road crossings, certain aspects of interactions between humans and social and industrial robots, especially mobile ones, apply. Similarly to AVs, it is crucial that mobile robots can co-exist and move safely among humans (Goodrich and Schultz, 2007). As highlighted by May et al. (2015), due to humans’ special needs to feel safe and comfortable when interacting with such robots, it is often not sufficient that robots treat humans merely as dynamic objects and try avoiding them. Instead, robots and humans need to have a mutual understanding of the situation and each other’s intentions. While humans often manage to assess the situation correctly based on the context and robot motion only (see, e.g., Lichtenthäler, 2014), several researchers posit that robots should apply non-verbal proactive communication to show their intentions to humans in the vicinity (Shattuck, 1995; Breazeal et al., 2001; Watanabe et al., 2015).

In communicating its intentions, a robot may describe both its goals (object or aim) and the reason for pursuing these goals (purpose). For example, May et al. (2015) investigated effects of showing navigational intent of a mobile robot to humans by means of (a) gaze and (b) a turn indicator. They found that both approaches had a significant effect; however, the approach (b) was more effective in communicating the intent and resulted in a higher perceived comfort. Similar results on human comfort are reported by Chadalavada et al. (2015) who explored robot intention communication via light projections in industrial scenarios. They also reported that the perceived level of comfort was for some conditions greater than the perceived comfort levels in the corresponding human–human interactions. In a similar study, Bunz et al. (2016) investigated the impact of projected intentions on human movement behavior where they did not find any significant effects. These findings were mainly motivated by shortcomings in the design of the communication interface (e.g., the projections were difficult to notice under some lighting conditions). Based on an online video survey involving animation of a social robot, Takayama et al. (2011) suggest that a robot showing its forethought before performing an action is more likely to be perceived as appealing, approachable, and sure of its subsequent actions. The ability to predict is stressed by Turnwald et al. (2016) who showed that humans are not only reacting, but also using prediction to plan their motion.

In summary, a robot providing humans with its intentions appears more reliable, predictable, and transparent to humans, which, in turn, facilitates trust that is a key for safety and comfort of humans involved in the interaction (Lee and See, 2004; Dautenhahn, 2007; Hoff and Bashir, 2014). Providing intention by means of motions might be insufficient in some situations. Translated to interaction between AVs and pedestrians, this indicates that communicating intentions of AVs to pedestrians in their vicinity might allow for a better understanding of the AVs and reduce ambiguities in communication due to lack of non-verbal communication with drivers. As highlighted by Bunz et al. (2016), it is, however, crucial that the communication interface is designed carefully. Finding the right balance between what, when, and how to communicate is one of the major challenges in human–robot communication design.

### Vehicle Design Solutions to Communicate With Pedestrians

To address communication issues between AVs and other road users in their vicinity, a few communication solutions making use of external vehicle interfaces have been suggested.

In 2012, a group of researchers at the Massachusetts Institute of Technology (MIT) suggested a biomimetic interface for automated and electric vehicles called AEVITA (Pennycooke, 2012). In the beginning of 2015, Mercedes demonstrated a concept vehicle that projects a zebra crossing in front of the pedestrian and issues auditory signal to cross the road (Mercedes-Benz, 2015). Later, Nissan showed a concept vehicle using a light strip around the vehicle and textual messages in the front windshield such as “after you” (Nissan News, 2015). Mitsubishi Electric has also revealed a concept where vehicle motion direction is projected onto the road surface (Mitsubishi Electric Corporation, 2015).

In addition, Google’s patent named Pedestrian Notification has recently been approved in the United States (USPTO, 2015). It uses, for instance, a flashing stop sign on the door of an AV to inform pedestrians not to cross the street in front of the car, or a robotic hand conveying various signals to them. Google has also added sound signals to their automated electric vehicles that mimic sound characteristics of conventional vehicles, and thereby alert pedestrians about their presence (Google, 2016).

It can be concluded that all these ideas are futuristic and/or brand-unique, and implementing them on other vehicles may not be trivial. Also, some of them are giving direct suggestions to pedestrians when to cross, which could in some situations be misleading (Andersson et al., 2017).

### Using an Artifact to Trigger Behavior

Since the 1980s, the research community started acknowledging that the context needs to be considered to understand cognition of individuals. One of the fundamental findings of cognitive engineering is that artifacts shape cognition and collaboration (Hutchins, 1995; Woods, 1998). When used in their context, artifacts trigger new behaviors. Prototypes of potential solutions thus function as tools for discovery to probe the interaction between people and technology to test the impact of new technological solutions in the context of human use in new envisioned contexts (Woods and Christoffersen, 2002). In our case, the envisioned context is how AVs and pedestrians can co-exist as agents in the traffic environment, and the AVIP interface is used as an artifact, or a tool, to trigger behaviors in that context.

## AVIP: Automated Vehicle Interaction Principle

### General Idea

The AVIP was created to evaluate the effect of AVs signaling their intent to pedestrians visually. Pedestrians’ needs for support in interactions with AV were derived based on: a literature review on current interactions between pedestrians and vehicles (see section “Background”), a field observational study in which interactions between pedestrians and vehicles, including AVs, were explored (see Malmsten Lundgren et al., 2017), a survey using photos with the same focus area as the field study, and expert board discussions (**Table 1**).

**Table 1:** Pedestrians’ support needs in interactions with AVs and derived functional requirements.

Pedestrians’ needs in interaction with AVs	Functional requirement
• Pedestrians should be able to easily distinguish if a vehicle is in manual or automated driving mode. This will keep the positive effect of eye contact in manually driven vehicles, and avoid possible dangerous situations due to a mismatch between the “driver’s” and the AV’s behavior.	Show when a vehicle is in automated driving mode
	
• Pedestrians need to obtain information about AVs future state (i.e., their intent and plans) rather than their current state. This, since the current state is directly observable from the vehicle’s movement, while the future state may be difficult to predict due to the lack of driver-centric cues.	Show future state of the AV
	
• Pedestrians should be provided with information that eliminates the need of seeking eye contact in encounters with AVs. This, since it may be difficult for them to deduce any accurate/useful information from the eye contact with the “drivers” in AVs.	Replace the eye contact
	
• Pedestrians should not be told explicitly when/where to cross the street in encounters with AVs. This, since other road users might pose a risk to the pedestrians that is not known by the AV.	Not urge pedestrians when/where to cross (i.e., just communicate the AVs intentions)
	
• Pedestrians should experience encounters with AVs as calm and not stressful. Calm pedestrians are more likely to make safe and predictable decisions.	Enable a calm interaction


To start with, seven low-fidelity interface concepts were developed, and then evaluated by an expert board consisting of representatives from both vehicle industry and academia. By considering a range of criteria including the technical and implementation feasibility, the board selected the most viable concept for further development and investigation: AVIP.

The underlying idea of AVIP is, instead of showing what pedestrians should do, to show pedestrians what the vehicle intends to do (Andersson et al., 2017). This is achieved by means of a minimalistic interface that could easily be used for any AV, independent of vehicle model and brand.

### The Visual Interface

**FIGURE 2:**
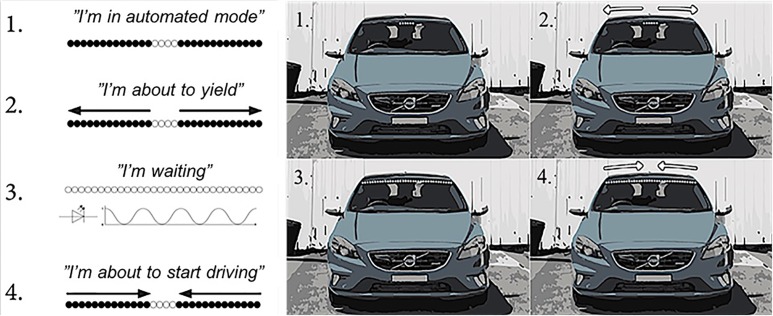
Schematic view of the four AVIP signals used in *Experiment I*. The “I’m waiting” signal was eliminated in *Experiment II*.

The essential part of AVIP is a light interface displaying visual signals to pedestrians at the top of the windshield. The signals were developed around four messages describing the vehicle mode and intent (**Figure 2**) that in turn were derived upon the needs specified in **Table 1**.

1. The “*I’m in automated mode*” signal is activated when the vehicle is operating in the automated mode, independently if there are some pedestrians in the vicinity. The signal is activated if the middle part of the interface is lit (**Figure 2.1**).
2. The “*I’m about to yield*” signal is initiated as soon as the AV intends to yield to someone. The signal can be canceled if the plan changes (e.g., if the pedestrian changes motion direction and walks away from the crossing). The signal is activated if the previous signal starts expanding toward the sides until the interface is completely lit (**Figure 2.2**).
3. The “*I’m waiting*” signal is initiated when the vehicle has stopped. The signal is activated if the interface is completely lit and pulsating slowly (**Figure 2.3**)
4. The “*I’m about to start driving*” signal is activated when the AV intends to continue driving (e.g., after yielding to a pedestrian). The signal is activated if the previous signal starts shrinking toward the middle of the interface (**Figure 2.4**).

It should be noted that initial concepts examined both auditory and visual interface modalities. External visual interfaces are commonly used for frequent communication from vehicles to other road users (e.g., turn indicator and brake light). Auditory interfaces, on the other hand, are used for less frequent, awareness raising signals (e.g., horn and emergency vehicle siren). Since interactions with pedestrians will be frequent, a visual interface using a light signal was used as the starting point for the design. Auditory signals are, however, possible to add at a later stage to create a multimodal interface, and thereby address some limitations with visual interfaces.

In addition, different signal characteristics such as frequency, area, color, and intensity have been explored. By changing the frequency and area of a signal, it is possible to make pedestrians aware of changes in the intention of AVs. In the traffic environment, several colors are already used for certain types of signals. To avoid a mix up, the following colors were excluded: red (prohibited to use in the front of the vehicle), green (strong connection to traffic signal light), blue (used by emergency vehicles, least suitable wavelength for human eyesight), and amber (used by service vehicles). Taking this into account, the choice was to use a white/yellow color for communicating all messages.

### The Implementation

**FIGURE 3:**
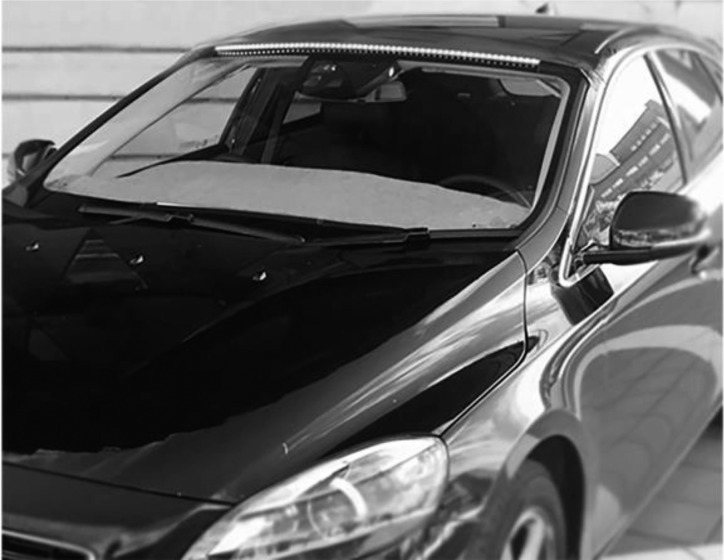
AVIP prototype installed in the test vehicle (Volvo V40).

To realize the concept and create a testable prototype, a 1-m RGB light-emitting diode (LED) strip was mounted outside the vehicle, at the top of the windshield (**Figure 3**). The LED strip was controlled via an Arduino microcontroller from inside the vehicle using a push button. Each press of the button triggers the next sequence in the signal loop (see **Figure 2**). The prototype was developed for installation in a Volvo V40 (**Figure 3**).

To emulate AVIP as an interface for AVs, a WOZ technique was applied. WOZ is a well-established approach for evaluating user interfaces in various domains, from robotics (Hoffman and Ju, 2014) to mobile applications (Carter and Mankoff, 2005) and automotive applications (Mok et al., 2015). It is based on the idea of simulating a fully working technical system by a human operator, a wizard (Steinfeld et al., 2009). It is often used to gather data from users who believe they are interacting with an automated system. However, in some cases, the users are informed about the wizard and his/her role.

**FIGURE 4:**
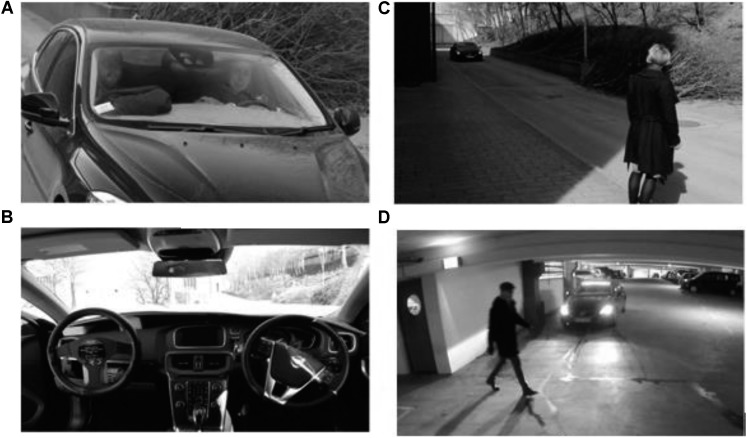
Exterior **(A)** and interior **(B)** of the test vehicle, and the test environment in *Experiment I* **(C)** and *Experiment II* **(D)**.

To create the WOZ setup, a dummy steering wheel was installed in a right-hand steered vehicle (a Volvo V40), and the real steering wheel was concealed. This way, it appeared to be a standard left-hand steered vehicle seen from pedestrians’ perspective (**Figure 4**). When the vehicle was operating in the manual mode, the fake driver on the left-hand side interacted with the pedestrians and seemingly drove the vehicle. When the vehicle was driven in the automated mode corresponding to SAE’s automation level 4 or 5 (SAE International, 2016), the fake driver on the left-hand side was reading newspaper.

This WOZ setup influenced our experimental design to some extent. For example, we had to make sure that the pedestrians did not take the opportunity to look directly into the vehicle when it is passing by. Otherwise, we would risk that they notice that the vehicle is only seemingly automated.

## Experiments: Approach And Results

To study how the interaction between pedestrians and AVs might look like in the future and how the interaction might be affected if AVs were to communicate their intent to pedestrians, we conducted two successive experiments in real-world traffic: *Experiments I* and *II*.

**FIGURE 5:**
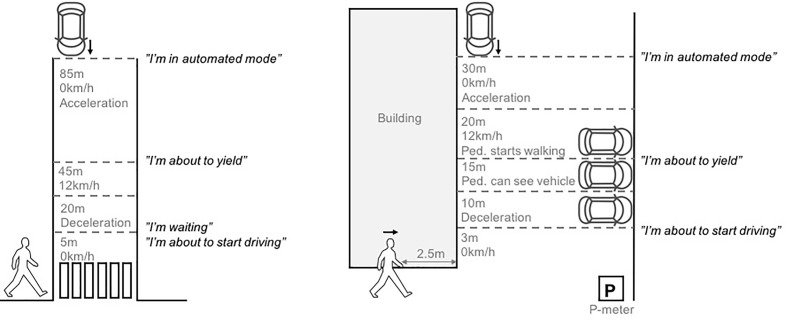
An illustration of the traffic situations investigated in *Experiment I* (left) and *Experiment II* (right). The approximate activation points of the AVIP signals are also shown.

The experiments involved a different number of pedestrians, and were conducted in two different traffic environments: a zebra crossing where vehicles are obliged to yield to pedestrians (*Experiment I*) and a parking garage where the yielding rules are less clear (*Experiment II*; see **Figure 5**). *Experiment I* provided an initial assessment of the understandability of the AVIP interface and how it may affect pedestrians’ perceived safety at a zebra crossing. It also informed the design of *Experiment II*.

In *Experiment I*, the pedestrians’ (un)willingness to cross the street and their self-assessed emotional reactions were used as an indicator of the perceived safety. To obtain a more direct measurement, the pedestrians in *Experiment II* were asked to assess their perceived safety by using a subjective rating scale. This proved to be a more time-efficient approach than the self-assessment of the emotions (that were rated in three dimensions; see section “Data Collection and Analysis”). Furthermore, *Experiment I* revealed a need to modify the AVIP interface (reduce the number of signals from four to three) to make it easier to interpret. Also, it revealed a need to control the experimental setting further to capture the critical moment of close interaction and ensure that the AVIP signals are activated at the same occasion in all encounters. Therefore, *Experiment II* was set up in conjunction to a garage wall corner to time the pedestrian’s arrival at the corner with the oncoming vehicle. An additional insight from *Experiment I* was that the familiarization phase is vital to eliminate potential “first encounter” effects. That is, the pedestrians were unfamiliar with both AVs and the AVIP interface, and they needed to gain at least some experience before the measurement of their perceived safety could start. *Experiment II* was hence designed with a long familiarization phase.

Another difference between the two experiments lies in the type of encounters that the pedestrians were exposed to. In *Experiment II*, the pedestrians were exposed to three types of encounters: (1) an AV with the AVIP signals, (2) an AV without the AVIP signals, and (3) a conventional vehicle. In *Experiment I*, the pedestrians were only exposed to (1) and (2). This choice was made mainly due to the time constraints, but also since the study by Malmsten Lundgren et al. (2017) already explored differences between the interactions with conventional vehicles and AVs (i.e., encounters 3 and 2) at the same location and using the same experimental approach.

The setup and results from *Experiment I* and *II* are further described in the following sections.

### Experiment I: Zebra Crossing

#### Purpose

The purpose of *Experiment I* was twofold: to assess pedestrians’ understandability of the AVIP signals in encounters with an AV at a zebra crossing and to explore pedestrians’ emotional experience and (un)willingness to cross the street as an indirect measure of their perceived safety. The focus was on investigating differences between encounters where the AV is communicating its mode and intent via the AVIP signals, and encounters without the AVIP signals. The findings and experimental insights informed the design of *Experiment II*.

#### Experimental Approach

To study understandability of the AVIP signals, the pedestrians were asked to observe the test vehicle from the pavement and report their interpretation of the signals to the test leader. To study their perceived safety, the study was set up as a “would cross/would not cross” experiment. That is, when a pedestrian encountered a vehicle, he/she was asked to assess whether he/she would cross the street. After each encounter, the pedestrians’ reasoning behind their (un)willingness was explored as well as their emotional experience.

#### Procedure

The experiment was carried out at a dead-end street at the Chalmers University of Technology (**Figure 4C**) and took about 40 min to complete. It was divided into three phases: familiarization (two encounters), understandability (four encounters), and comparison (two encounters)^1^.

In the familiarization phase, the test leader informed the pedestrian about the experiment. It was revealed that he/she would encounter an AV, but AVIP and its function were not mentioned. The pedestrian then filled in a consent form and a background questionnaire, and the task was explained before he/she took a given position at the curb (ca 0.5 m from the roadway). The pedestrian was asked to imagine that he/she was standing near a marked zebra crossing. First, the pedestrian was facing the test leader (with his/her back toward the approaching vehicle to ensure that all pedestrians start observing the vehicle at the same distance and speed). The vehicle started moving approximately 85 m from the pedestrian and moved along a straight road section. When the vehicle was approximately 50 m from the pedestrian, the test leader asked him/her to turn around to observe the approaching vehicle. The vehicle was at that point in time moving at a speed of approximately 12 km/h, and with the AVIP signal “*I’m in automated mode*” active (see **Figure 2**). When the vehicle was approximately 45 m from the pedestrian, the AVIP signal “*I’m about to yield*” was activated. When the vehicle was approximately 20 m from the pedestrian, it started decelerating and eventually came to a full stop ca 5 m from the pedestrian. At that time, the AVIP signal “*I’m waiting*” was activated. After a short while, the AVIP signal “*I’m about to start driving*” was activated and the vehicle started to slowly accelerate. Next, the pedestrian was asked to turn toward the test leader and to elaborate on his/her experience in the given encounter to find out if the prototype was noticed, and how it was interpreted (the pedestrian was asked to turn his/her back toward the road to avoid seeing the interior of the vehicle and the WOZ setup through the side window). Following this, the intended function of the AVIP interface was explained, and then its signals were demonstrated in a second encounter (same vehicle speed and timing of the AVIP signals as in the previous encounter).

In the understandability phase, the pedestrian was asked to look toward the approaching AV with the AVIP prototype and to interpret its signals. At the test leader’s indication, the pedestrian turned around toward the test leader (with his/her back toward the roadway) to elaborate on the signals that the AVIP was showing. The pedestrian was also asked to rate how confident he/she was regarding the interpretation on a 1–5 scale, where 1 is not sure and 5 is very sure. In one encounter type, the pedestrians started to observe the vehicle when it was approximately 85 m from him/her, and the indication to turn around was given when the vehicle was at approximately 45 m distance and had a speed of approximately 12 km/h (assessing the “*I’m in automated mode*” and “*I’m about to yield*” signals). In another encounter type, the pedestrian started observing the vehicle when it was standstill and turned around when the vehicle was about to accelerate again (assessing “*I’m waiting*” and “*I’m about to drive*” signals). These two encounters were experienced in a randomized order twice (i.e., four encounters in total).

In the comparison phase, the pedestrian was asked to look toward the approaching vehicle (the same speed and timing of the AVIP signals as in the previous phases) and to assess if he/she would have started to cross the street before the vehicle had stopped and why (without actually crossing the street). Each pedestrian encountered the AV with and without the AVIP prototype in a randomized order. After, the pedestrian was asked to compare his/her experiences meeting the AV with/without the prototype.

#### Participants

To participate in the study, the pedestrians had to be familiar with the test location and frequently travel by foot. In total, nine pedestrians were recruited (five male, four females, mean age interval: 20–29 years) through direct contact at the Chalmers University of Technology. According to Nielsen and Landauer (1993), a usability test of a product involving nine participants discovers over 95% of the usability problems. This discover rate was considered good enough for an initial prototype evaluation. As for the assessment of the perceived safety, the data are used to obtain an initial indication about how pedestrians may feel rather than statistical evidence.

#### Data Collection and Analysis

The data collected in the first two phases consisted of a background questionnaire, pedestrian’s statements regarding the AVIP signals, and their confidence when assessing them. In the comparison phase, the pedestrians were asked to indicate whether they would cross the street or not, to complete a Self-Assessment Manikin (SAM) questionnaire (Bradley and Lang, 1994), and to answer questions in a semi-structured interview about their experiences in the given encounter. SAM is a non-verbal assessment method that measures the valence, activity, and control associated with a person’s affective reaction to stimuli.

#### Results

The WOZ approach was successful in making all pedestrians involved in the experiment (*N* = 9) believe that they were experiencing an AV.

In the familiarization phase, eight out of nine pedestrians noticed the AVIP, but they failed to interpret its function. However, after the short training, the pedestrians were more successful in the interpretation of the AVIP signals. More specifically, seven out of nine pedestrians succeeded in interpreting the “*I’m in automated mode*” signal. The rest reported that they were confused about who was in control of the vehicle. All pedestrians successfully interpreted the other three signals. In addition, the pedestrians felt confident in their interpretations of the signals. The median value of their interpretation confidence on the 1–5 confidence scale was: 5 for the “*I’m in automated mode*,” 4 for the “*I’m about to yield*,” 4 for the “*I’m waiting*,” and 5 for the “*I’m about to drive*” signals.

**FIGURE 6:**
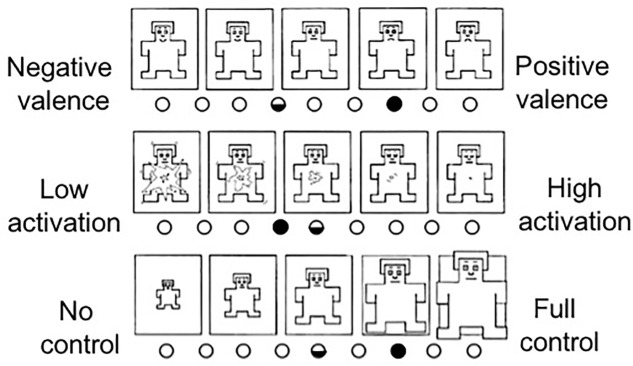
The average SAM rating scores of nine pedestrians in *Experiment I* regarding valence, activation, and control with the AVIP signals (full dot) and without the signals (half dot).

In the comparison phase, the pedestrians encountered the AV twice: once without the AVIP signals and once with the signals. The findings showed that the AVIP slightly increased the pedestrians’ willingness to cross the road before the vehicle came to a full stop. When encountering the AV without the AVIP, only one out of nine pedestrians stated that they would start crossing before the vehicle stopped completely. This number increased to three out of nine when the AVIP signals were activated. Regarding the emotional experiences with and without the AVIP signals, the following can be concluded based on the SAM ratings (**Figure 6**):

• On average, the pedestrians rated the valence as more positive with the AVIP signals activated.
• On average, the pedestrians rated their activation level as slightly lower with the AVIP signals activated.
• On average, the pedestrians felt more in control of the traffic situation when the AVIP signals were activated.

In other words, the AVIP prototype affected the pedestrians’ emotional experience positively; they felt calmer when encountering the AV with the AVIP signals. This is also supported by their statements in the interview at the end of the experiment. For example, several pedestrians stated that they were missing the AVIP signals when they were not activated. Three of the pedestrians stated, for example:

“*When the vehicle was driving itself without the prototype, the situation became very weird. It is like I am losing all control. But with the prototype, when you get used to it, it is very clear… I really want to keep it.*”

“*It felt better than the last time because now I can see what the car thinks.*”

“*With the prototype, I could understand much more of what was about to happen. I trust the car more.*”

Interestingly, several pedestrians stated that the “*I’m waiting*” signal was not contributing to their experience, and that it could be removed to make the interface easier to understand. To exemplify, three of them stated:

“*It looked like the car was in a kind of idle mode, but it didn’t tell me much at all.*”

“*It was in fact a bit difficult to notice if the signal was pulsating or not. The other signals were easy to distinguish and interpret.*”

“*The pulsating signal was not that clear…and even if you notice the pulse, it is a bit tricky to interpret it.*”

Another reoccurring theme in the interviews was that this type of interface could, with some more experience, provide more accurate information than when looking at the drivers today. One of them stated, for example:

“*I looked first at the lights and then at the driver, it was quite intuitive, I must say. I knew that I would get more information from the lights than from watching the driver in this case.*”

Also, a majority of the pedestrians reported that it was easy to get used to the prototype and that the trust toward it would increase with more experience. Two of them stated:

“*Now I have learned to recognize the signal. I think that, with a little more experience, I could probably interpret it much faster than I did now.*”

“*Now when I’ve started to get used to it, it feels more natural… It really tells you when something is happening.*”

### Experiment II: Parking Garage

#### Purpose

The purpose of *Experiment II* was mainly to study pedestrians’ perceived safety. In contrast to *Experiment I* where the perceived safety was derived from pedestrians’ (un)willingness to cross the street and the SAM questionnaire, the perceived safety was here directly assessed by the pedestrians. The SAM questionnaire was omitted because it can be time consuming, and it gives only an indirect assessment of the perceived safety. The pedestrians commented on their emotions in the interview. Also, the pedestrians were exposed to encounters with a conventional (manually operated) vehicle and with an AV (with/without AVIP), and the encounters took place in a parking garage where the interactions occurred in closer proximity than at the zebra crossing. An additional difference compared to *Experiment I* was that the “*I’m waiting*” signal in the AVIP interface was excluded. This choice was made since results from *Experiment I* showed that removing the signal could make the interface easier to understand.

#### Experimental Approach

The study was set up as a controlled experiment of crossing a road in a public parking garage. The focus was on pedestrians’ perceived safety as well as their emotional experience in the encounters with a conventional vehicle and an AV.

#### Procedure

The experiment was carried out in a parking garage at Lindholmen Science Park, Gothenburg. The total time of the experiment was approximately 20 min. The experiment was executed in four blocks (*A–D*) consisting of two, three, four, and eight encounters, respectively (**Table 2**). The first three blocks (*A–C*) were carried out for training purposes, while the last block (*D*) resulted in the experiment data that are analyzed and elaborated upon in this paper.

**Table 2:** An example of the encounters for one pedestrian.

			Vehicle motion profile				
			*AVIP signals*				
			
Block	Encounter	Driving mode	Before pedestrian becomes visible	Pedestrian becomes visible	Pedestrian crosses toward the parking meter	Pedestrian at the parking meter	Pedestrian crosses toward the starting point
A	1	MD	Approaching	Approaching	Standstill	Standstill	Standstill
	2	MD	Standstill	Standstill	Standstill	Passing by	–
B	3	AD	Motion	Approaching	Standstill	Passing by	–
	4	AD	Standstill	Standstill	Standstill	Standstill	Standstill
	5	AD	Passing by	–	–	–	–
C	6	AD w. AVIP	Approaching	Approaching	Standstill	Standstill	Standstill
			*I’m in automated mode*	*I’m about to yield*	*I’m about to yield*	*I’m about to yield*	*I’m about to yield*
	7	AD w. AVIP	Standstill	Standstill	Standstill	Passing by	–
			*I’m in automated mode*	*I’m about to yield*	*I’m about to yield*	*I’m about to start driving*	
	8	AD w. AVIP	Passing by	–	–	–	–
			*I’m in automated mode*				
	9	Demonstration AD w. AVIP	Approaching	Approaching	Standstill	Passing by	–
			*I’m in automated mode*	*I’m about to yield*	*I’m about to yield*	*I’m about to start driving*	
D	10	MD	Approaching	Approaching	Standstill	Standstill	Standstill
	11	AD w. AVIP	Passing by	–	–	–	–
			*I’m in automated mode*				
	12	AD	Standstill	Standstill	Standstill	Standstill	Standstill
	13	AD w. AVIP	Standstill	Standstill	Standstill	Standstill	Standstill
			*I’m in automated mode*	*I’m about to yield*	*I’m about to yield*	*I’m about to yield*	*I’m about to yield*
	14	MD	Standstill	Standstill	Standstill	Standstill	Standstill
	15	AD	Approaching	Approaching	Standstill	Standstill	Standstill
	16	AD w. AVIP	Approaching	Approaching	Standstill	Passing by	–
			*I’m in automated mode*	*I’m about to yield*	*I’m about to yield*	*I’m about to start driving*	
	17	AD	Standstill	Standstill	Standstill	Standstill	Standstill


*MD, manual driving; AD, automated driving; AD w. AVIP, automated driving with active AVIP signals. The encounters in *Blocks A–C* were conducted for training purposes and in the same order for all pedestrians, while the encounters in *Block D* were randomized and resulted in the data that are analyzed in this paper.*

Prior to the encounters, the test leader informed the pedestrian that he/she would interact with a test vehicle, without mentioning that the vehicle could be automated. The pedestrian then filled a consent form and a background questionnaire, and together with the test leader took a given position approximately 2.5 m from the roadway (behind a building wall, see **Figure 5**). The pedestrian was told to imagine a situation where he/she was about to pay for a parking ticket and needed to cross the road, pay for the ticket at the parking meter, and return to the starting point. To enable timed and repeatable interactions between the vehicle and the pedestrian, the test leader had continuous phone contact with the driver.

In *Block A*, the pedestrian encountered a manually operated vehicle (i.e., the fake driver was seemingly driving the vehicle and seeking eye contact with the pedestrian, while the vehicle was operated by the person in the right-hand seat). In the first encounter, the vehicle started accelerating ca 30 m from the building corner and the place where the pedestrian would later cross the road (see **Figure 5**). When the vehicle reached a constant speed of approximately 12 km/h (approximately 20 m from the building corner), the test leader indicated to the pedestrian to start walking toward the parking meter. At approximately 10 m, the vehicle started braking and eventually stopped approximately 3 m from the building corner. The vehicle remained still until the pedestrian crossed toward the parking meter and returned to the starting point behind the wall. The pedestrian decided on his/her own when it was safe to cross toward the parking meter, and when it was safe to return. In the second encounter, the vehicle was first at a standstill approximately 3 m from the building corner. When the pedestrian reached the parking meter, the vehicle passed by. After each encounter, the test leader asked the pedestrian the following: (a) “*How did you experience the encounter with the vehicle?*” and (b) “*On a scale 1–5, where 1 is unsafe and 5 is safe, how safe did you feel in this encounter?*”

*Blocks B* and *C* were carried out in a similar manner. However, after stopping to yield to the pedestrian, the vehicle passed without waiting for him/her to return from the parking meter to the starting point. Also, a third encounter for which the vehicle passed by before the pedestrian reached the roadway was added to *Block B* to reduce the feeling that the vehicle would always stop. With this, the vehicle that the pedestrian encountered could have one of the following three motion profiles: (a) approaching with a speed of approximately 12 km/h and then decelerating to a full stop, (b) passing by with a speed of ca 12 km/h, and (c) standing still with a speed of approximately 0 km/h. In both *Blocks B* and *C*, the pedestrian encountered an AV. However, in *Block C*, the AVIP was activated. First, it was showing the “*I’m in automated mode*” signal. When the pedestrian was visible around the corner, the “*I’m about to yield*” signal was activated. The “*I’m about to start driving*” signal was activated when the pedestrian reached the parking meter. After three encounters in *Block C*, the test leader asked the pedestrian if he/she had noticed something unexpected, and explained that the vehicle was sometimes operated in the automated mode (without mentioning the WOZ approach). Also, the test leader asked if the pedestrian noticed the AVIP signals, and in that case, how these were interpreted. The test leader then explained the idea behind the signals, followed by an additional encounter where the signals were demonstrated.

*Block D* was carried out in a similar manner as *Blocks C* and *B*. However, in addition to the encounters with the AV (with and without AVIP), the pedestrian encountered a conventional vehicle as well. To avoid the pedestrian “automating” his/her behavior, the vehicle remained still in some of the encounters until the pedestrian crossed toward the parking meter and returned to the starting point behind the wall. All encounters were executed in a random order.

#### Participants

To participate in the study, the pedestrians had to be adults (18+). In total, 24 pedestrians were recruited (12 males, 12 females, mean age interval 18–30 years) through direct contact, social media, and e-mail.

#### Data Collection and Analysis

The data collected consisted of a background questionnaire, pedestrians’ statements describing their experience in the encounters with the test vehicle and pedestrians’ self-assessed level of perceived safety on a 1–5 Likert scale (1 is unsafe, 5 is safe). The answers were directly annotated on a paper sheet as well as audio recorded. The perceived safety levels were grouped according to the vehicle state (manual driving, automated driving, and automated driving with AVIP) and vehicle motion pattern (standstill, passing by, and approaching). A Mann–Whitney *U*-test (Sheskin, 2000) was conducted to determine if there are any significant differences in the pedestrians’ perceived safety for any of these combinations of vehicle state and motion pattern. In addition, a qualitative assessment of the pedestrians’ statements was also performed to identify common themes (Strauss and Corbin, 1998).

#### Results

The experiment conducted in the parking garage shows results that are in line with the results from *Experiment I*. To start with, all pedestrians (*N* = 24) believed that they were interacting with an AV (i.e., they did not suspect that they were exposed to a WOZ setup). Further, the AVIP signals were noticed under the training phase (*Blocks A–C*) by a majority of the pedestrians. However, just a few of them related the signals to the vehicle’s mode and intention. This was not surprising as the general public is still largely uneducated about AVs and is unaware about the technical development. After a short explanation about AVs and the AVIP signals, a frequent comment from the pedestrians was that the interface is in fact easy to interpret.

**FIGURE 7:**
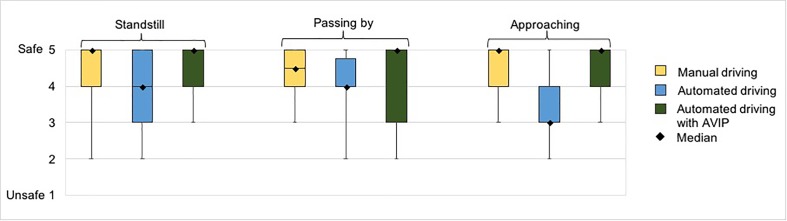
Whisker diagram with median values (black squares) of the pedestrians’ perceived safety in *Experiment II* on the rating scale 1–5, where 1 is unsafe and 5 is safe.

This ease of interpretation was also reflected in subjects’ judgment of safety in the encounters with the standstill vehicle (see **Figure 7**). The Mann–Whitney *U*-test indicated that the pedestrians’ perceived safety (self-assessed on a rating scale 1–5) was significantly greater in the encounters with the standstill conventional vehicle (*Mdn* = 5) than in the encounters with the standstill AV (*Mdn* = 4); *U* = 9.908, *p* = 0.002, α = 0.05. However, there was no significant difference in their perceived safety between the encounters with the conventional vehicle (*Mdn* = 5) and the encounters with the AV with the AVIP signals (*Mdn* = 5); *U* = 0.229, *p* = 0.632, α = 0.05. On the other hand, the pedestrians felt safer in the encounters with the standstill AV with the AVIP signals (*Mdn* = 5) than in the encounters with the standstill AV (*Mdn* = 4); *U* = 8.358, *p* = 0.004, α = 0.05. That is, in the standstill encounters, the pedestrians felt equally safe when encountering the conventional vehicle and the AV with the AVIP signals, while they felt less safe in the corresponding encounters with the AV without the AVIP signals.

The positive effect of the AVIP signals was even more emphasized when considering the pedestrians’ safety rating in the encounters with the vehicle in motion. According to the Mann–Whitney *U*-test, the pedestrians’ perceived safety was significantly greater in the encounters with the approaching AV with the AVIP signals (*Mdn* = 5) than in the encounters with the approaching AV (*Mdn* = 3); *U* = 19.416, *p* = 0.000011, α = 0.05. Similarly, the pedestrians’ perceived safety was significantly greater in the encounters with the approaching conventional vehicle (*Mdn* = 5) than in the encounters with the approaching AV (*Mdn* = 3); *U* = 18.643, *p* = 0.000016, α = 0.05. However, there was no significant difference in their perceived safety in the encounters with the approaching conventional vehicle (*Mdn* = 5) and the encounters with the approaching AV with the AVIP signals (*Mdn* = 5); *U* = 0.0000; *p* = 1; α = 0.05. This suggests that the AVIP signals increased the pedestrians’ perceived safety to the same level as they experienced in the encounters with the approaching conventional vehicle.

The impact of the AVIP signals was, however, less apparent for the encounters where the vehicle passed by before the pedestrian reached the roadway. Although there is a slight difference in the medians of the pedestrians’ perceived safety (see **Figure 7**), the Mann–Whitney *U*-test suggests that these differences are not significant. As motivated by the pedestrians, these encounters were not associated with any notable risk since the vehicle cleared the roadway before it became a threat, and they felt rather safe independently of how the vehicle was operated and if the AVIP signal was activated or not.

The analysis of the pedestrians’ statements showed that the lower level of perceived safety in encounters with the AV was mainly motivated by the lack of non-verbal communication with the driver. Three of the pedestrians stated:

This was unpleasant, I did not know if it has noticed me at all. I waited until I made sure it had completely stopped.

I know that the vehicle is intelligent, it was slowing nicely but still it did not feel convincing.

The vehicle was standing still and I could not figure out if it would accelerate or not. I looked at the driver but it did not help. It is not only about eye contact, it is the entire behavior of the driver that I usually look at.

On the contrary, the level of safety in the encounters with the conventional vehicle was mainly explained by clear communication from the driver. Several pedestrians highlighted that the eye contact with the driver is important, but also that they paid attention to both the driver and vehicle behavior: “*vehicle was smoothly slowing down, and I could see the driver clearly*,” “*driver looked at me, she was not going to accelerate*,” “*first I looked at the vehicle, then I double-checked with the driver*.”

The pedestrians commonly explained that the AVIP signals made it easier for them to assess the situation: “*showed clearly it has noticed me*,” “*easy to see it will stop*,” “*I knew it would wait*.” The signal dynamics (e.g., changing from “*I am in automated mode*” to “*I am about to yield*”) was appreciated, and several pedestrians mentioned that this gave them an indication that sensors are working and/or that they have been noticed: “*almost like having eye contact with the driver*.” However, one of the pedestrians was concerned that the dynamics may be difficult for elderly people to notice and interpret.

## Discussion

### Insights on Interactions With Automated Vehicles

Currently, there are only a few studies that have investigated issues that may arise when highly AVs are introduced in our traffic alongside conventional vehicles (see section “Interactions between automated vehicles and pedestrians”). To shed light on this topic, we have conducted two experiments (*Experiments I* and *II*) exploring interactions between pedestrians and AVs in two different traffic situations: a zebra crossing and a parking lot.

Overall, our findings suggest that the interactions with such vehicles may be different from interactions with conventional vehicles today and that the role of non-verbal communication may be different. In *Experiment I*, the pedestrians commonly stated that they experienced a low level of valence and control, at the same time as they experienced a high level of satisfaction, in encounters with the AV. Although the pedestrians did not encounter any conventional vehicles in *Experiment I*, they commonly related their experiences with the AV in the experiment to the corresponding encounters with conventional vehicles in everyday life, pointing out that they were missing interaction with the driver and to get “acknowledgment” that they have been noticed. Several of the pedestrians both in *Experiments I* and *II* highlighted that they were previously unaware of how much they rely on communication with drivers. Some also stated that they were looking for the driver and observing the driver behavior even when they knew the vehicle was in the automated mode. In *Experiment II*, pedestrians experienced a lower level of safety in encounters with the AV compared with the conventional vehicle. Together, these studies indicate that pedestrians are likely to experience a feeling of stress and unsafety when encountering an AV, implying that current interaction patterns and strategies with conventional vehicles cannot be directly transferred to AVs. Similar findings were reported in our previous study (Malmsten Lundgren et al., 2017) as well as by Merat et al. (2016) and Böckle et al. (2017).

These findings are not surprising, as pedestrians are currently not used to interact with highly AVs. As demonstrated in Section “Interactions Between Conventional Vehicles and Pedestrians,” the process of interaction between pedestrians and conventional vehicles is today not completely understood. It is a complex process that involves a combination of factors such as: prior experience, background knowledge about the traffic environment and interactions in that environment, observation of vehicle movements and other relevant characteristics, and beliefs and knowledge of formal rules and social norms. One important part of the process is thought to be a series of cognitive processes that allow people to estimate the internal mental states and to predict future actions of others (Sebanz et al., 2006). In close proximity encounters, such as zebra crossings and parking lots, the cognitive processes involved are highly based on non-verbal communication with drivers; by interpreting cues in a driver’s behavior such as eye contact and gestures, pedestrians can reliably predict their intentions and the near-term movements of the vehicle (Schmidt and Färber, 2009; Sucha et al., 2017). By contrast, when it comes to assessing the imminent actions of AVs, pedestrians do not currently possess comparable intuitive abilities. In AVs, the person sitting in the driver’s seat is not responsible for the vehicle control and his/her behavior may not necessarily reflect the behavior of the vehicle. This makes it difficult for pedestrians to assess the encounters with AVs and, unless AVs are equipped with technical means that enable them to reveal their “mental states” and intentions to pedestrians in the vicinity, pedestrians are likely to experience discomfort and unsafety in encounters with such vehicles.

### The Role of the AVIP Signals

Inspired by the research in human–robot interaction where it is shown that revealing intentionality of robots makes them more predictable and generally more appealing to humans (see section “The Value of Showing Intentions”), we designed a visual interface (AVIP) able to communicate the mode and intentions of AVs to pedestrians (and other road users) in their vicinity.

Overall, our findings suggest that an external vehicle interface that communicates the AV’s mode and intent to pedestrians, such as AVIP, may create a positive experience in the interaction; it may increase pedestrians’ perceived safety and make them feel calm. The results from *Experiment II* indicate that it could, in fact, increase pedestrians’ perceived safety to the same level as they experience in encounters with conventional vehicles. Based on our results, it is, however, difficult to pinpoint with certainty if a higher level of perceived safety will lead to a higher level of actual safety. On the other hand, previous studies indicate that it might be the case; calm people are more likely to make better-informed decisions (Forgas, 1995; Mather and Lighthall, 2012; Resnick, 2012). This is an issue open for further research.

In some situations, pedestrians interpret a conventional vehicle’s intention without any non-verbal communication with the driver (e.g., in darkness or in poor visibility where we cannot see the driver’s face or gestures). As argued by some contemporary studies (e.g., Rothenbucher et al., 2016; Risto et al., 2017), the intent of AVs could perhaps be sufficiently communicated based on motion patterns only. Still, our results indicate that communicating the intent of the AV using an external interface can contribute to a positive interaction experience. Learned positive experiences are important for building trust and acceptance toward new technologies (Hoff and Bashir, 2014). Recent surveys show that people are currently largely skeptical toward AVs (Heinkel, 2017), and if an AV can give pedestrians a feeling of trust and can enable a common understanding of what is about to happen, this should be a strong advantage for any AV manufacturer.

One should also remember that AVs may unintentionally send confusing and misleading signals to pedestrians through their motion. Imagine that an AV slows down prior to turning at an intersection. Such an action could cause a nearby pedestrian to incorrectly believe that the vehicle is slowing down since it intends to yield to him/her. This, and similar vehicle actions, could thus lead to safety-critical decisions. The risk of misleading signals through vehicle motion could be reduced by means of an interface such as AVIP that clearly shows when an AV intends to yield.

As with any unfamiliar technology, when AVs start entering the market, pedestrians (and other road users) in their surroundings may not necessarily have assumptions or beliefs that are aligned with the actual capabilities of these vehicles. They may underestimate, for instance, the object detection abilities of AVs, and consequently become too uncertain and cautious when interacting with such vehicles. This could, in turn, lead to deadlocks, safety problems, and traffic inefficiency. Similarly, there may be occasions when pedestrians overestimate capabilities of AVs, and behave in a risky manner due to incorrect assumptions (e.g., that AVs can always stop). The fact that AVs from different manufacturers are likely to differ somewhat in their capabilities may make it even more difficult for pedestrians to correctly assess capabilities of AVs. While we have not assessed these issues in our experiments, we believe, based on the general idea of the AVIP, that these and similar issues may be mitigated if AVs are able to clearly show what they are about to do next. As such, showing intentions externally may be viewed as a way to reveal the actual capabilities of AVs.

At the same time, these issues call for harmonization, or even standardization, of external communication principles. To avoid confusion, it is necessary that such communication is unified across different vehicle types and brands. However, the questions what should be unified, and to what extent, remain. One design principle that we applied for the AVIP, and that we believe should be adopted independently of the interface implementation, is to communicate intent rather than explicitly invite people to act. This is to avoid possible ambiguities due to a mismatch between the vehicle’s invitation and the surrounding traffic (Andersson et al., 2017). Furthermore, when asked if they would like AVs to signal something else, the pedestrians in our experiments agreed that showing the mode and yielding intentions is necessary and satisfying. Also, the dynamics of the signals was especially appreciated since it indicated a change in the intent, implying that such design principles may be worth considering in the harmonization process.

### Limitations of the Study

One of the major limitations of our studies goes back to the fact that AVs (and AVIP) do not exist in traffic today, and that our test participants (pedestrians) do not have any previous experience of such vehicles (and interfaces). To account for this, and reduce the “first encounter” effect, our experiments included a familiarization phase consisting of multiple encounters. After a few encounters, the pedestrians commonly commented that they are becoming used to the new type of encounters, indicating that the experiment design was successful in making them feel somewhat familiar with AVs. However, it is still unknown how interactions between pedestrians and AVs may develop over time, and how it may be reflected in their needs to be informed about the mode and future intentions of AVs.

An interesting, and somewhat surprising, finding was that a great majority of the pedestrians stated that they believed that the vehicle was in automated mode when the person behind the (fake) steering wheel was reading the newspaper. While this suggests that the WOZ approach was successful in simulating automated driving, a real AV would have enabled a more flexible experimental setup (e.g., to reduce the risk that a pedestrian see the vehicle interior, it was necessary that the vehicle passes by when the pedestrian is not facing the road).

Given that this field is in its infancy, there are currently no standardized tools for assessing interactions with AVs. In *Experiment I*, we used the SAM to assess pedestrians’ emotions. However, some pedestrians reported difficulties in understanding the control scale, and we noticed that assessing emotions in three dimensions could be time consuming. Also, this approach provided only an indirect measure of a pedestrian’s perceived safety. In *Experiment II*, a five-item Likert scale was used instead to obtain a direct assessment of the pedestrians’ perceived safety. While this tool was easy to use, the future studies may gain from using complementary tools that, for instance, measure pedestrians’ biometrics.

Furthermore, our studies involved a limited number of participants in Sweden, and we believe that a larger and more heterogeneous sample of participants is needed to be able to draw more solid conclusions. In *Experiment I*, the pedestrian participants were mainly students at the Chalmers University of Technology, and they are not necessarily representative of the general population as they are generally younger and may also have a higher affinity toward technology. To eliminate such issues, *Experiment II* included a more heterogeneous sample of participants. However, it is still not unlikely that the experiment attracted a certain type of people. Future studies should thus be of a more naturalistic character. It is also vital to take into consideration cultural differences in pedestrian behavior, and how these differences may be manifested in interactions with AVs.

### Future Work

Further studies (yet to be published) explore the value of intent communication in more dynamic traffic situations and for other road users at the testing track AstaZero in Sandhult, Sweden. In particular, interactions between AVs and conventional vehicles are explored in the context of symmetrically narrowed roads where yielding traffic rules are unclear similar to a parking lot setting. Another area that we are exploring is if the intent communication of automated truck platoons could improve interaction with manually operated vehicles (e.g., to prevent conventional vehicles to cut in between automated trucks in a platoon). In future studies, it would also be advantageous to investigate interactions with AVs in real-world traffic to address some of the topics that were highlighted previously such as the relationship between the intent communication and motion patterns of the vehicle, and pedestrian behavioral changes over time.

## Conclusion

Taking the two experiments together, it can be concluded that pedestrians may need support to experience safe interactions with AVs. Particularly, they may need means to replace information that they today obtain from drivers via non-verbal cues in low-speed situations where negotiations are needed.

To examine if such information could be replaced by a minimalistic external interface, we suggested an interface that communicates to pedestrians whether or not an AV is in the automated mode and what the vehicle intends to do next. Due to safety reasons, the interface does not instruct pedestrians when and where to cross the road. The idea is that the interface should not interfere with the vehicle design. In other words, the interface is not linked to a certain vehicle model nor brand. Instead, it could be deployed across different brands in a standardized way.

The interface was utilized in a WOZ setup under naturalistic conditions in two different traffic situations. The results showed that the pedestrians could, after a short training course, understand the signals conveyed by the interface, and that they were confident in their interpretation of these signals. In both traffic situations, the pedestrians reported that the interface replaced the role of the non-verbal cues that they obtain from drivers (e.g., eye contact), and contributed to a higher level of perceived safety and more pleasant interactions with AVs.

Our studies imply that communicating the mode and intent of AVs via simple external interfaces could be sufficient to improve interactions between pedestrians and AVs by creating a higher perceived safety for pedestrians. However, we suggest further investigations in more dynamic traffic situations and involving a larger number of pedestrians to validate this conclusion and to determine how it relates to vehicle motion patterns. It should also be noted that any type of additional external signaling on vehicles may require standardization to avoid potential ambiguities.

## Risk And Ethics Assessment

Prior to each of the experiments, RISE Viktoria (independent, governmentally owned, and non-profit research institute) conducted a regular internal risk and ethics assessment. In both cases, it was concluded that all potential risks are eliminated and that the experiments are in line with good research ethics. That is, the participants are not exposed to any larger risk than when being in regular daily traffic (their task is to cross the street if/when they feel safe and to assess their experience). The participants are informed about their task and the experiment process in advance, both in written and oral form. The participants are not informed in advance about the WOZ approach where the experiment vehicle appears to be operated by automation, but is in fact operated by a licensed human driver. However, they are informed about the WOZ approach after the completed experiment. This is to avoid affecting their experiences when interacting with (seemingly) AVs. Due to the nature of the experiments, it was concluded that a further assessment by the regional Ethical vetting is not needed.

## Author Contributions

AH and VML contributed to interface development, experiment design (*Experiments I* and *II*), experiment execution (*Experiments I* and *II*), analysis (*Experiments I* and *II*), and writing. JA contributed to experiment design (*Experiment II*), experiment execution (*Experiment II*), analysis (*Experiment II*), and writing. MK contributed to interface development, experiment design (*Experiment I*), and writing. TL contributed to interface development, experiment design (*Experiment I*), experiment execution (*Experiment I*), and analysis (*Experiment I*). AS, JF, CE, RF, SK, DS, and PL contributed to interface development, experiment design (*Experiment I*), and manuscript review.

## Conflict of Interest Statement

CE was employed by company Volvo Cars Group. RF was employed by company Autoliv AB. SK was employed by company Scania AB. DS and PL were employed by company Volvo Group AB. The remaining authors declare that the research was conducted in the absence of any commercial or financial relationships that could be construed as a potential conflict of interest.

## Footnotes

1 See the **Supplementary Material** for more information.
