# Supplementary material for: Communicating Intent of Automated Vehicles to Pedestrians
Source: Front Psychol. 2018 Aug 7;9:1336. doi: 10.3389/fpsyg.2018.01336 (PMC6090516; doi:10.3389/fpsyg.2018.01336)
Supplement: Supplementary file 1 [file Table_1.pdf]

Supplementary Material

Communicating intent of automated vehicles to pedestrians

Azra Habibovic*, Victor Malmsten Lundgren, Jonas Andersson, Maria Klingegård, Tobias Lagström, Anna Sirkka, Johan Fagerlönn, Claes Edgren, Rikard Fredriksson, Stas Krupenia, Dennis Saluäär, Pontus Larsson

*** Correspondence:** Azra Habibovic, azra.habibovic@ri.se

1 Supplementary Tables

Supplementary Table 1. Procedure in the *Experiment I.*

Phase	Encounter #	AVIP signal(s)	Narrative
FAMILIARIZATION	1	*I'm in automated mode*<br>-> *I'm about to yield*<br>-> *I'm waiting*<br>-> *I'm about to start driving*<br>-> *I'm in automated mode*	• Vehicle accelerates from a ca 85 distance while pedestrian is turned with his/her back towards the roadway. The (fake) driver is reading newspaper. The *I'm in automated mode* signal is activated.<br>• Pedestrian turns towards the roadway and starts observing the vehicle (12 km/h) when it is ca 50 m from him/her.<br>• At ca 45 m distance, *I'm about to yield signal* is activated.<br>• At ca 20 m distance, the vehicle starts decelerating.<br>• The vehicle stops at ca 5 m distance and *I'm waiting* signal is activated.<br>• After ca 30 seconds, the *I'm about to start driving* signal is activated.<br>• The *I'm in automated mode* signal is activated and the vehicle starts accelerating.<br>• Pedestrian turns his/her back towards the roadway and answers the following questions:<br>○ *Describe what you saw?*<br>○ *How did you experience the traffic situation?*<br>○ *How did you experience the light signals in the windshield?*<br>○ *What function did the light signals in the windshield have?*
	2	Same as Encounter #1	Same as Encounter #1, but different questions:<br>○ *Do you have any questions about the system?*<br>○ *Have you understood how the system works?*<br>○ *Do you have any comments about interacting with a self-driving car and its attempt to communicate with you?*
UNDERSTANDABLY	3	*I'm in automated mode*<br>-> *I'm about to yield*	• Vehicle accelerates from a ca 85 distance while pedestrian is turned with his/her back towards the roadway. The (fake) driver is reading newspaper. The *I'm in automated mode* signal is activated.<br>• Pedestrian turns towards the roadway and starts observing the vehicle (12 km/h) when it is ca 50 m from him/her.<br>• At ca 45 m distance, *I'm about to yield* signal is activated.<br>• Pedestrian turns his/her back towards the roadway and answers the following questions:<br>○ *What signal did the car show?*<br>○ *How confident are you on your answer on a scale from 1 to 5 where 1 is uncertain and 5 is very certain?*
	4	*I'm waiting*<br>-> *I'm about to start driving*<br>-> *I'm in automated mode*	• The vehicle stops at ca 5 m distance and *I'm waiting* signal is activated.<br>• After ca 30 seconds, the *I'm about to start driving* is activated.<br>• The *I'm in automated mode* signal is activated and the vehicle starts accelerating.<br>• Pedestrian turns his/her back towards the roadway and answers the questions (same as in Encounter #3).
	5	Same as Encounter #3	Same as Encounter #3
	6	Same as Encounter #4	Same as Encounter #4
COMPARSON	7	Same as Encounter #1	Same as Encounter #1
	8	Automated driving, no AVIP	Same as Encounter #1 (but without AVIP signals active)
